# Description of network meta-analysis geometry: A metrics design study

**DOI:** 10.1371/journal.pone.0212650

**Published:** 2019-02-20

**Authors:** Fernanda S. Tonin, Helena H. Borba, Antonio M. Mendes, Astrid Wiens, Fernando Fernandez-Llimos, Roberto Pontarolo

**Affiliations:** 1 Pharmaceutical Sciences Postgraduate Programme, Federal University of Paraná, Curitiba, Brazil; 2 Research Institute for Medicines (iMed.ULisboa), University of Lisbon, Lisbon, Portugal; 3 Department of Pharmacy, Federal University of Paraná, Curitiba, Brazil; 4 Department of Social Pharmacy, Faculty of Pharmacy, University of Lisbon, Lisbon, Portugal; Humanitas University, ITALY

## Abstract

**Background:**

The conduction and report of network meta-analysis (NMA), including the presentation of the network-plot, should be transparent. We aimed to propose metrics adapted from graph theory and social network-analysis literature to numerically describe NMA geometry.

**Methods:**

A previous systematic review of NMAs of pharmacological interventions was performed. Data on the graph’s presentation were collected. Network-plots were reproduced using Gephi 0.9.1. Eleven geometric metrics were tested. The Spearman test for non-parametric correlation analyses and the Bland-Altman and Lin’s Concordance tests were performed (IBM SPSS Statistics 24.0).

**Results:**

From the 477 identified NMAs only 167 graphs could be reproduced because they provided enough information on the plot characteristics. The median nodes and edges were 8 (IQR 6–11) and 10 (IQR 6–16), respectively, with 22 included studies (IQR 13–35). Metrics such as density (median 0.39, ranged 0.07–1.00), median thickness (2.0, IQR 1.0–3.0), percentages of common comparators (median 68%), and strong edges (median 53%) were found to contribute to the description of NMA geometry. Mean thickness, average weighted degree and average path length produced similar results than other metrics, but they can lead to misleading conclusions.

**Conclusions:**

We suggest the incorporation of seven simple metrics to report NMA geometry. Editors and peer-reviews should ensure that guidelines for NMA report are strictly followed before publication.

## Introduction

Network meta-analysis (NMA) is an increasingly attractive statistical method used to compare all treatments of interest in a given condition [1, 2], by simultaneously synthesizing data from direct and indirect evidence [3, 4]. Like all statistical modeling, NMA has a number of assumptions that should be satisfied to avoid erroneous results and misleading conclusions [5, 6]. The first assumption is that all direct evidence is connected in a network of comparisons, which can be checked by building a plot [7, 8].

Graph drawing, as part of the mathematical concept of graph theory, has extensively been used in many research disciplines, such as social network analysis, electrical networks, biology experimental designs, and chemistry [9–12]. This technique allows for the modeling of pairwise relations among a set of objects, and is useful to ground judgmental and analytical decisions from a macro view of results [13–15]. In the field of NMA, a conventional network graph consists of ‘‘nodes” representing the interventions of interest and “edges” representing available direct comparisons between pairs of interventions [16, 17]. The amount of evidence can also be presented by ‘‘weighting” the nodes and edges with different node sizes and line thicknesses [18, 19]. This graphical display allows a wider visualization of NMA’s available evidence, which may help to guide the initial interpretation of the results for rational clinical decisions [20–22].

However, considering that similar NMA structures may present different numbers of nodes, edges and included studies, it is challenging to judge only by a graphical display where one provides more valuable evidence. Thus, the use of special measures for benchmarking and proper interpretation of data is paramount in a detailed graph analysis. These measures, called metrics, are defined as a set of graph properties converted into a rational number. Several metrics with distinct properties are available [23–26] and could be used to describe the geometry of NMAs and highlight their strengths and weaknesses, regardless of the size or structure similarity of the networks, or even been used when the absence of the network plot itself.

The PRISMA extension statement for reporting of systematic reviews incorporating network meta-analyses of health care interventions (PRISMA-NMA) was designed to improve the completeness of reporting NMA data [16, 17]. This checklist includes three items on NMA geometry. In the methods section, item S1 File proposes the description of the methods used to explore the geometry of the network, including information on graphical summary. In the results section, items S3 and S4 recommend, respectively, the presentation of the network structure and a brief overview of the characteristics of the network geometry that may include the number of trials and patients involved, and evidence gaps [16]. Despite these recommendations and the recent development of software statistics for NMA conduct [27, 28] with different tools for building network-plots [29–32], limited guidance exists on how best to present NMAs in an accessible format, especially for non-technical end-users, such as policymakers and clinicians [33, 34]. Conversely, for pairwise meta-analysis presentations, where standards for displaying forest-plots are commonly used [35, 36], no established or standardized metrics for reporting NMA geometry exist [37]. Thus, we aimed to propose simple adapted parameters and metrics from the social network analysis literature and test their usability to describe the geometry of NMA plots.

## Methods

### Literature search and eligibility

A systematic review was performed according to the PRISMA statement and Cochrane Collaboration recommendations [38, 39]. Further information on the systematic review were previously published [40].

Two reviewers performed all of the steps of the systematic review process (i.e. title and abstract reading (screening), full-text appraisal and data extraction) individually, and discrepancies were resolved by a third author (PRISMA checklist–S4 File).

Searches were conducted in two scientific literature database platforms (PubMed and Scopus), without limits for time-frame or language (update April 25th, 2017). A manual search in the reference lists of included studies and grey literature searches (Google) were also performed. The full search strategies are in supplementary material (S1 File). We included studies reporting NMAs (e.g. multiple or mixed treatment comparisons/meta-analysis, indirect meta-analysis) comparing any drug therapy intervention head-to-head or against placebo. We considered any type of network (open or closed-loops) of experimental, quasi-experimental, or observational trials. Non-NMAs, study protocols, studies reporting data only on non-pharmacological interventions, and articles written in non-Roman characters were excluded.

### Data extraction, metrics proposal and testing

We used a standardized data collection form to extract data on: (i) the study general characteristics (authors names, countries of affiliation, publication year) and (ii) network key-aspects: presence of network-plot (graphical representation of comparisons) and description of the geometry, including number of nodes (i.e. interventions), number of edges (i.e. direct comparisons evidence), and number of included studies (thickness of the edges).

The network-plots of all included NMA studies were replicated using Gephi 0.9.1 (https://gephi.org/). The network-plot is defined as a graph (G), an ordered pair of nodes (N) or vertices, together with a set of edges (E) or lines. After the replication of NMA plots, we applied eleven adapted descriptive parameters and geometry metrics from previous concepts of social network analyses and graph theory to describe all NMA structures [23–26]. The definition of the adapted parameters and metrics are shown in Table 1 (see S2 File for metrics to describe NMAs).

**Table 1:** Metrics definition.

Parameter or metric	Definition*
Number of nodes	Total number of interventions represented as nodes (vertices) of the network (graph)
Number of edges	Total number of direct comparisons between the nodes of the network, referred to as edges or lines
Number of studies	Total number of studies included in the network for each direct comparison or edge
Average degree	The degree of a node is the number of edges incident to the node, with loops counted twice. The total degree of a graph is the sum of the degree of all nodes. The average degree is a network level measure. It is calculated from the value of degree of all nodes in the graph, divided by the number of nodes.
Average weighted degree	A graph is a weighted graph, if a number is assigned to each edge. In this case, the weight is the number of studies per edge. The weight of the graph is the sum of the weights given to all edges, divided by the total number of nodes.
Density	Density is a measure of the connectedness of a graph, and is defined as the number of connections, divided by the number of possible connections. The graph is dense if the number of edges approaches the maximal number of edges possible (value closer to 1.0), otherwise is sparse (value closer to 0).
Percentage of common comparators±	Complete graphs have the feature that each pair of nodes has an edge connecting them. In this case, all nodes are directly linked and can be considered ‘common comparators’. The higher the percentage of common comparators, the more strongly connected is the network. Different from what may occur with density, this metric may better represent the visual display of a network.
Percentage of strong edges±	The number of studies in an edge is proportional to the existing direct evidence among two nodes. Edges with only one study can be considered a weak piece of the network. Strong edges contribute more to the robustness of the evidence. This metric accounts for the percentage of edges with more than one study (named ‘strong edges’).
Mean thickness±	The thickness of an edge is the number of studies assigned to that edge. The mean thickness of a graph is the total number of studies, divided by the total number of edges. This can be obtained by the division of the average weighted degree by the average degree. However, it does not consider the dispersion of the values.
Median thickness with dispersion value±	Different from the mean thickness, the median thickness is the expression of the median number of studies per edge in a network, along with a dispersion measure reported as interquartile ranges (IQR 25% and 75%).
Average path length	The length of a path is the number of edges that a path uses to reach node to node. The average path length is the number of steps along with the shortest paths for all possible pairs of nodes in the network.

*All parameters and metrics were adapted from previous studies on social network analysis and graph theory [23–26].

±Metrics especially created to support the report of NMAs geometry.

### Metrics’ statistical analyses and sensitivity

Descriptive analyses were conducted with all parameters and metrics. Variable normality was assessed with the Kolmogorov-Smirnov Test and re-evaluated through Q-Q normal plots that revealed that all variables that were non-normally distributed. The variables were then expressed as absolute and relative frequencies.

To test the usability of the eleven proposed parameters and metrics to describe the NMA geometry, we compared the results obtained for each parameter and metric among all the evaluated networks and performed sensitivity analyses including:

- Comparison of the results obtained for each parameter and metric among networks with different structures, i.e. visual display (geometry), but with the same number of nodes and edges;
- Comparison of the results obtained for each parameter and metric among networks with equal structures, i.e. visual display (geometry), but different number of included studies.

Considering the results obtained during the sensitivity analyses, and to explore the relationship between all the eleven proposed parameters and metrics, the Spearman test for non-parametric correlations was used. The Bland-Altman plot and Lin’s concordance test (concordance correlation coefficient) were used to analyze the agreement between the metrics presenting a moderate-strong correlation. Thus, the aim of these correlation analyses was to evaluate the level of association among metrics and to avoid reporting overlap (i.e. that is, metrics measuring the same characteristic). The parameters and metrics that presented better results during the analyses, identified as relevant to describe NMA geometry, were selected for discussion. All analyses were conducted in IBM SPSS Statistics v. 24.0 (Armonk, NY: IBM Corp.) and probabilities below 5% were considered statistically significant [41–43].

## Results

**Fig 1:**
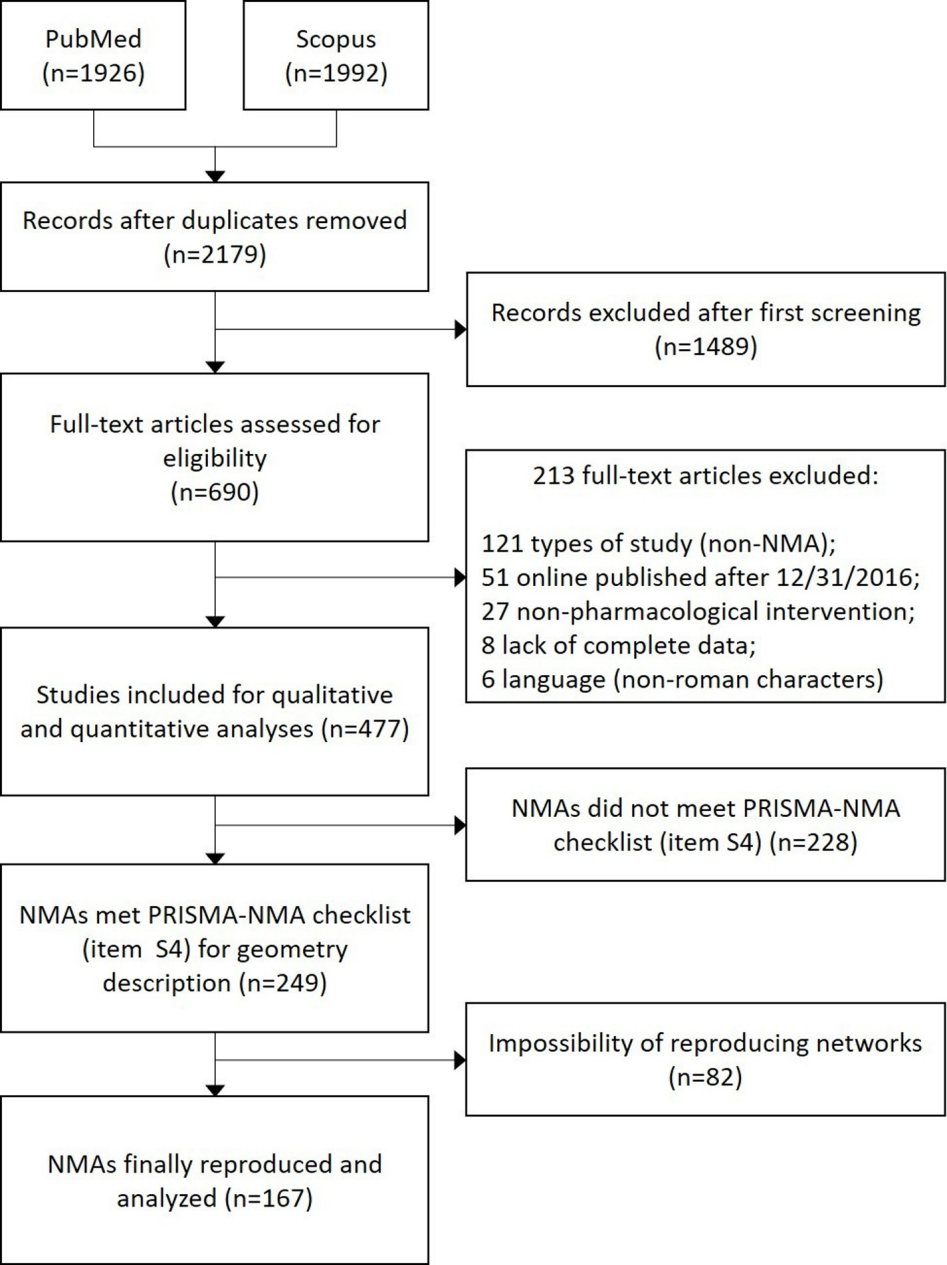
Flowchart of the included NMAs for network-plot reproduction and geometry assessment.

The systematic search in PubMed and Scopus yielded 2179 registers, of which 690 were fully appraised and a total of 477 NMAs were considered for the analyses. The display of the network-plot (item S3 from PRISMA-NMA statement) was provided by 79.4% of these NMAs, but a minimum set of descriptions of the network geometry were presented, according to PRISMA-NMA item S4, by only 249 studies (52.2%). However, during the replication of the network-plots, just 167 NMAs (35.0%) provided enough information about the graph geometry that allowed its reproduction (e.g. data on the number of studies for each edge). See Fig 1 for the flowchart of this process.

The overall results of the geometry of the 167 NMAs, after applying the eleven proposed parameters and metrics, is shown in Table 2. The full-assessment of each NMA is in the Open Science Framework platform (doi: 10.17605/OSF.IO/SP7UM). Overall, the included networks had a median of 8 ‘nodes’ (IQR 6–11) and 10 ‘edges’ (IQR 6–16) with 22 included ‘studies’ (IQR 13–35).

**Table 2:** Assessment of NMAs geometry.

Descriptive analyses (n = 167 NMAs)	N. of nodes	N. of edges	N. of studies	Avg. degree	Avg. weight degree	Density	Common comparator %	Strong edges %	Mean thickness	Median Thickness	Avg. path length
Mean	8.83	12.0	30.23	2.63	7.98	0.43	68.0	53.0	2.95	2.17	1.73
SD	5.10	8.49	29.32	0.82	7.3	0.23	26.0	30.0	2.42	1.77	0.47
Median	8.00	10.00	22.00	2.55	5.67	0.39	7.3	55.0	2.18	2.0	1.69
IQR 25	6.00	6.00	13.00	2.00	3.50	0.26	50.0	29.0	1.50	1.0	1.50
IQR 75	11.00	16.00	35.00	3.00	9.33	0.53	89.0	75.0	3.54	3.00	1.89
Minimum	3.00	3.00	3.00	1.50	1.57	0.07	9.0	0.0	1.00	1.00	1.00
Maximum	42.00	66.00	157.0	5.14	50.00	1.00	100.0	100	20.00	13.00	5.25
Asymmetry ± error	2.75 ±0.19	2.52 ±0.19	2.31 ±0.19	0.94 ±0.19	2.63 ±0.19	1.01 ±0.19	-0.52 ±0.19	-0.02 ±0.19	3.33 ±0.19	3.12 ±0.19	2.77 ±0.19

N.: number; Avg: average; SD: Standard deviation; IQR: interquartile range; %: represented as percentage

The ‘average degree’ (degree of connection) of the networks was of 2.55 connections per node (IQR 2.00–3.00). A total of 6 networks presented the lowest value for this metric (1.50), with all of them composed by 4 nodes and 3 edges. The highest ‘average degree’ (5.14 edges per node) was obtained for a network with 7 nodes and 18 edges. The mean ‘percentage of common comparators’ (nodes with more than one connection) was around 70%, with 38 plots considered strongly connected (100% of nodes as common comparators). Around 35% of networks presented half of their nodes with only one connection (‘loose-ends’). The ‘density’ (total number of connections in the network divided per the number of possible connections) varied from 0.07 for the most poorly connected network (32 nodes and 32 edges) to 1.0 in 12 completely connected networks (e.g. structures with 3 nodes and 3 edges; 4 nodes and 6 edges; 5 nodes and 10 edges). The ‘average path length’ of the networks (distance between nodes) was 1.69 (IQR 1.50–1.89), varying from 1.00 for small networks (e.g. 3 nodes and 3 edges; 4 nodes and 6 edges) to 5.25 in large networks (plot with 32 nodes and 32 edges).

The overall ‘mean thickness’ of the evaluated networks was of 2.95 studies per edge. One small network (4 nodes, 5 edges) with 119 trials reached the highest value for this metric (20.00 studies per edge), that corresponded to 13.00 studies per edge (IQR 8.00–34.00) considering the metric ‘median thickness’. Eleven networks presented only one study per edge, while 23 networks (plots varying from 3 nodes and 3 edges to plots with 8 nodes and 14 edges) presented all edges (100%) with more than one study (‘percentage of strong edges’ metric).

**Fig 2:**
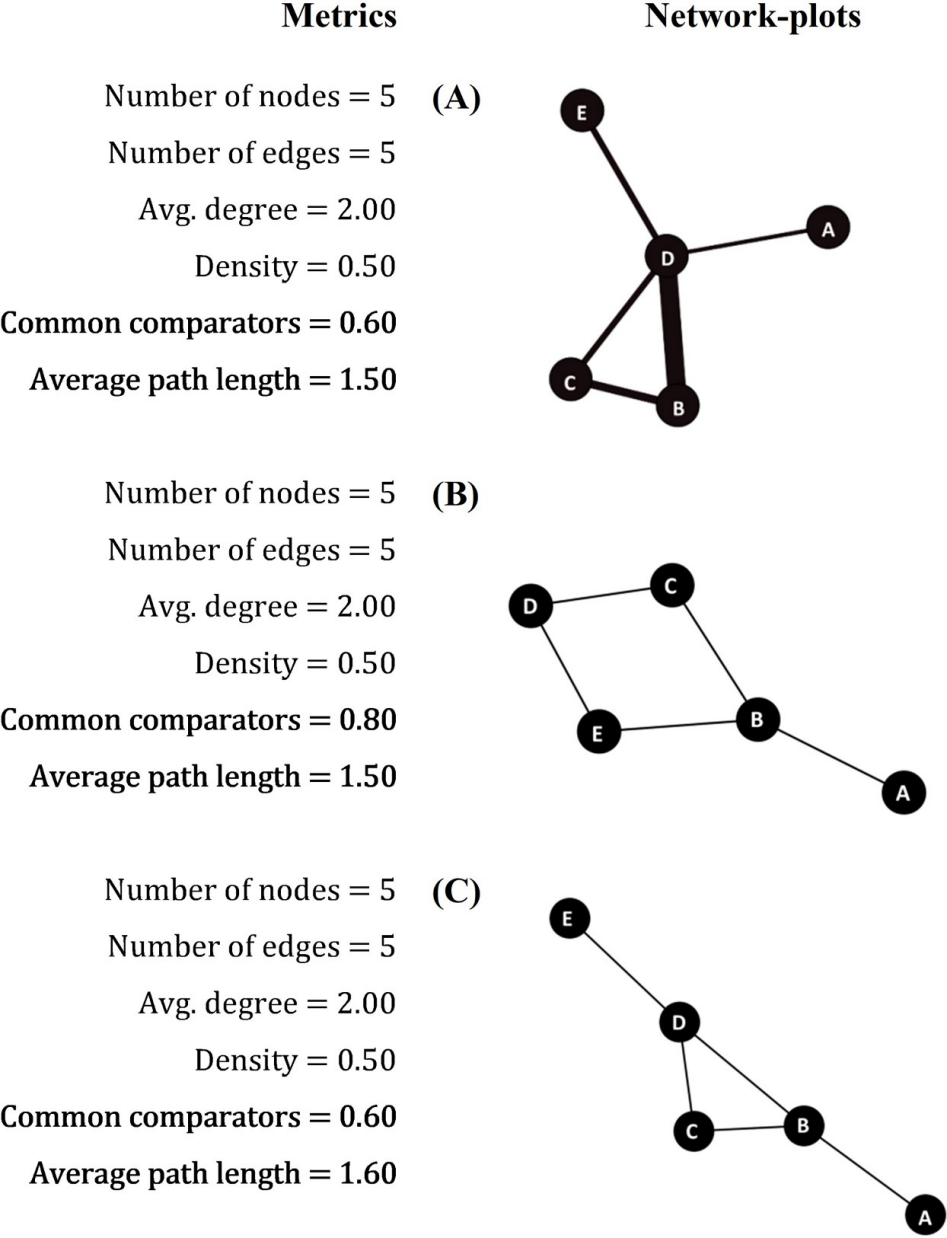
Sensitivity analyses for the assessment of geometry of NMAs with similar number of nodes and edges. Examples of three networks-plots from the 167 analyzed NMAs. Highlighted parameters showed different values among similar NMAs.

The sensitivity analyses highlighted some differences in the metric’s results for networks with equal number of nodes and edges, but with different three-dimensional structures (graph display). We have exemplified these differences in Fig 2, using three NMAs included in the systematic review (named as A, B, C) that present identical size, with 5 nodes and 5 edges, because they were the most frequently reported among the 167 NMAs with graphs provided. Since the total number of included studies in all of these three networks was 5, this variable was not considered in this first sensitivity analysis. ‘Density’ and ‘average degree’ values were equal between the three network plots (0.5 and 2.00, respectively). However, differences were noted in the metric ‘percentage of common comparators’, where networks with more loose-ends (nodes with only one connection) have lower rates of ‘common comparators’ (60% for networks A and C; 80% for network B). The ‘average path length’ also differed among these networks, but with a different pattern than the other metrics, with values of 1.50 for structures A and B, and 1.60 for graph C.

**Fig 3:**
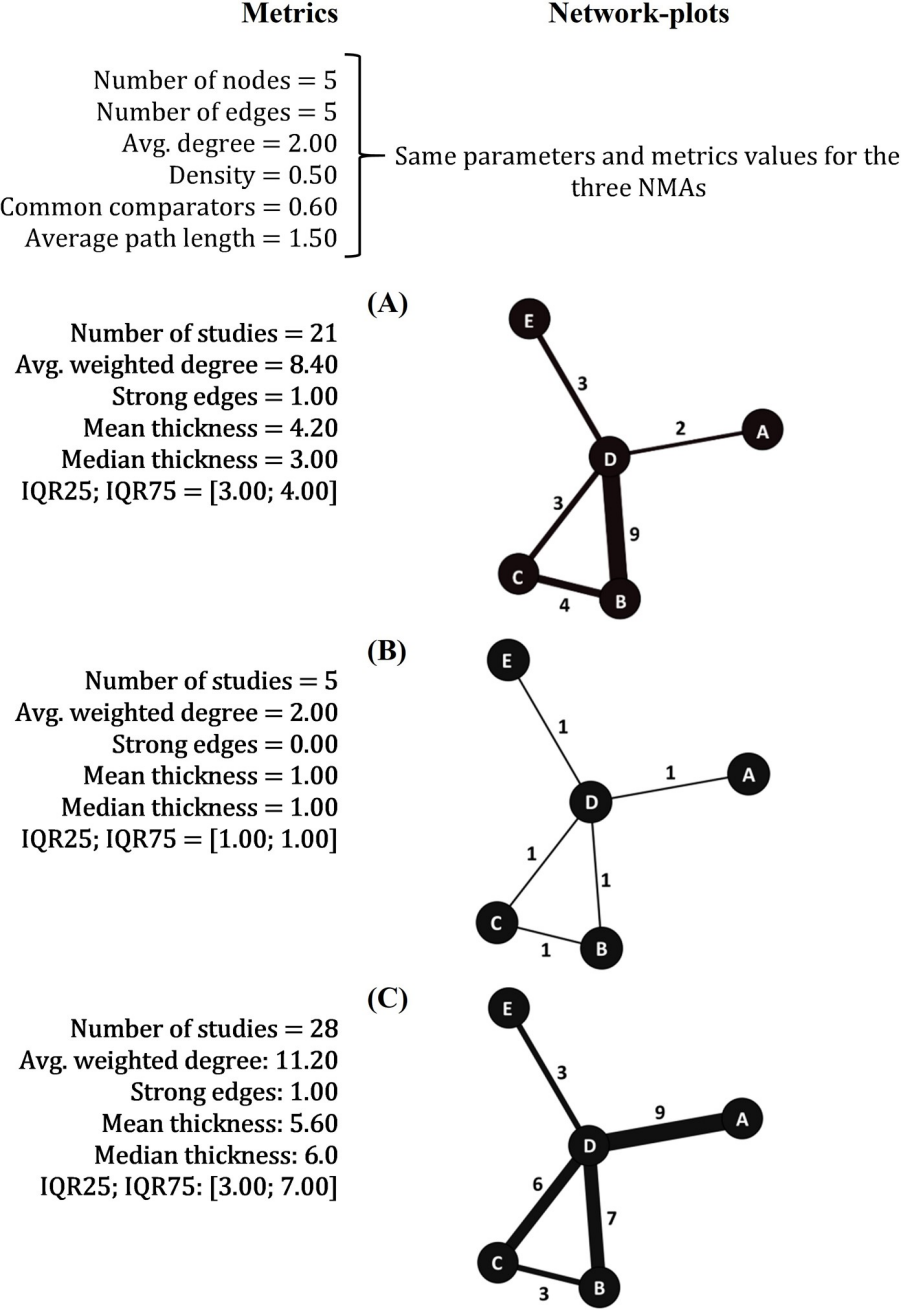
Sensitivity analyses for the assessment of NMAs with equal geometry and different numbers of studies. Examples of three networks-plots from the 167 analyzed NMAs. Highlighted parameters showed different values among similar NMAs.

Sensitivity analyses also revealed different metric results for networks with equal geometry structures, but with different numbers of included studies (Fig 3). We have also exemplified this analysis with three similar plots (A, B, C) from our systematic review. In this case, differences were noted in the weight of evidence. ‘Average weighted degree’, ‘mean thickness’, and ‘median thickness’ showed similar performances, presenting higher values in networks with more studies. Network-plot B presented weaker evidence than networks A or C, with only one study per edge (0% of ‘strong edges’; ‘mean thickness’ = 1.00). Network-plot C presented an ‘average weighted degree’ of 11.20 with a median of 6.0 studies per edge [IQR 3.00–7.00] while network A showed a median of 3.0 studies per edge [IQR 3.00–4.00].

Analyses revealed some strong positive correlations as between ‘average weighted degree’ and ‘mean thickness’ (Spearman’s ρ 0.907; p<0.001) and between ‘mean thickness’ and ‘median thickness’ (Spearman’s ρ 0.865; p<0.001). ‘Percentage of common comparators’ also correlated with ‘density’ (Spearman’s ρ 0.626; p<0.001). Negative, but strong, correlation was found for ‘percentage of strong edges’ with ‘average weighted degree’ (Spearman’s ρ -0.732; p<0.001), with ‘mean thickness’ (Spearman’s ρ -0.867; p<0.001), and with ‘median thickness’ (Spearman’s ρ -0.903; p<0.001) (see Table 3). However, the concordance analyses and Bland-Altman plots showed that ‘mean’ and ‘median thickness’ were the only metrics to present substantial agreement (concordance correlation coefficient ρ_c_ = 0.820) (see S3 File).

**Table 3:** Correlation analyses of NMA’s geometry parameters and metrics.

Correlation Spearman’s Rho (n = 167 NMAs)	N. of nodes	N. of edges	N. of studies	Avg. degree	Avg. weight degree	Density	Common comparator	Strong edges	Mean thickness	Median thickness	Avg. path length
N. of nodes		**0.886**	0.437	0.285	-0.170	**-0.827**	-0.209	0.425	-0.323	-0.430	**0.736**
p-value		**<0.001**	<0.001	0.022	0.028	**<0.001**	0.007	<0.001	0.001	<0.001	**<0.001**
N. of edges			**0.585**	**0.662**	0.113	-0.490	0.163	0.305	-0.165	-0.294	0.416
p-value			**<0.001**	**<0.001**	0.145	<0.001	0.035	0.002	0.035	<0.001	<0.001
N. of studies				**0.505**	**0.674**	-0.130	0.220	-0.352	0.540	0.359	0.080
p-value				**<0.001**	**<0.001**	0.093	0.004	0.001	<0.001	<0.001	0.300
Avg. degree					0.503	0.264	**0.741**	-0.012	0.129	0.033	-0.270
p-value					<0.001	<0.001	**<0.001**	0.877	0.096	0.669	<0.001
Avg. weight. degree						0.473	0.494	**-0.732**	**0.907**	**0.754**	-0.482
p-value						<0.001	<0.001	**<0.001**	**<0.001**	**<0.001**	<0.001
Density							**0.626**	-0.441	0.424	0.473	**-0.918**
p-value							**<0.001**	<0.001	<0.001	<0.001	**<0.001**
Common comparator								0.157	0.233	0.186	**-0.560**
p-value								0.042	0.020	0.016	**<0.001**
Strong edges									**-0.867**	**-0.903**	-0.427
p-value									**<0.001**	**<0.001**	<0.001
Mean thickness										**0.865**	-0.423
p-value										**<0.001**	<0.001
Median thickness											-0.464
p-value											<0.001
Avg. path length											
p-value											

N.: number; Avg: average. Bold values show moderate-very strong and statistically significant correlation between metrics.

## Discussion

Our evaluation of the geometry of 167 NMA plots indicates that the description of some parameters and metrics are crucial to ensure network reproducibility and may help during evidence interpretation, especially because these network plots are readers’ first contact with the available evidence. We have adapted and tested the usability of eleven metrics for NMA geometry description, grounded on social network analysis and graph theory literature.

Until recently, NMAs were only used by researchers with a strong statistical background, but the development of user-friendly software has popularized this method [2, 4]. However, there is a series of conceptual challenges when conducting and reporting a NMA and these should also be considered by clinicians who read such scientific publications [3, 6]. Firstly, the presentation of NMA results is not as straightforward as in traditional pairwise meta-analysis [22, 44]. The validity of NMA is based on the underlying assumption that there is no imbalance in the distribution of effect modifiers across the different types of direct treatment comparisons, regardless of the structure of the network [8, 45].

Previous studies showed that the synthesis methods and analytical processes for NMA conduct and reporting, including the representation of network structure and other diagrams, still need improvement [46, 47]. Improvement is also necessary because network structures also seem to have influence on the final results of NMAs. Salanti and collaborators have investigated 18 different NMAs [20] and showed that entirely star shaped networks (or close to this pattern) have only one comparator, typically placebo or no active treatment. This pattern may suggest study treatment preference bias (e.g. publication bias, missing outcome data), with strong or ubiquitous avoidance of head-to-head comparisons of active treatments [21, 48], and should be carefully interpreted.

In our analyses, we were able to reproduce only 35% of the NMA-plots found in the systematic review. Part of this issue was due to the lack of a network diagram or minimum description of geometry, as recommended by the PRISMA-NMA statement. Another group of network-plots, although minimally complying with the PRISMA-NMA checklist items, failed to detail some information about the graph (e.g. amount of studies included in each edge) that prevented their replication. This highlights the need to review the PRISMA-NMA checklist to standardize the report of NMA, requiring authors to provide a minimum set of parameters and metrics of geometry to allow reproducibility.

As we have shown by replicating the network-plots, the graphical presentation of the network provides an accessible and understandable format for describing the evidence, how information flows indirectly, the contribution of certain interventions, and the evidence gaps [37, 49]. Usually, the more treatments and studies included in a network, the more clinically informative the NMA is [49]. However, large networks informed by few studies often yield imprecise evidence and may show inconsistencies, whereas a smaller network contains less evidence but may show no clear inconsistencies [50, 51]. For this reason, the network graph itself is not enough to provide a complete and transparent picture of the available evidence. Slight modifications in the NMA geometry may also have impact on the evidence resulting from the analysis and subsequently influence the decision making process. Thus, in addition to network size, the description of parameters and metrics is useful to supplement graph information [20], especially for distinguishing similar NMAs, as we have demonstrated in our sensitivity analyses. Moreover, a proper geometry description can foster the statistical analysis of the NMA, help in procuring reliable estimates and recommend further trials if necessary [37, 49].

After testing eleven metrics, we suggest that, besides reporting three obvious items (number of ‘nodes’, ‘edges’ and ‘number of studies per edge’), four additional metrics should be incorporated in the future NMA report: ‘density’, ‘percentage of common comparators’, ‘median thickness’ (median of number of studies per edge with interquartile ranges) and ‘percentages of strong edges’. ‘Density’ is a measure of the connectedness in a graph, revealing how many edges are needed to complete the network [13, 14]. However, in two different NMAs with the same number of nodes and edges, density is identical. This measure is not influenced by the network three-dimensional display and does not depend on the size of the network. In this case, a complementary measure–the ‘percentage of common comparators’–was useful for better defining the display of the structure. This metric provides the number of loose-ends (nodes with only one connection) in the network, which represent poorly compared interventions in the literature that should be better investigated in future trials.

On the other hand, the results of ‘average path length’ were found to be misleading. This metric is commonly used in social network analyses to account for the distance between objects in the network [14, 24]. However, for NMAs, the average distance between all of the interventions does not correspond to the number of loose ends or missing edges in the network. Networks with the same number of nodes, edges, and loose ends may have different ‘average path length’ that vary according to structure.

Among the measures evaluating the ‘weight’ of evidence, we found that ‘average weighted degree’ may also be misleading, since its results are double of those obtained by ‘mean thickness’. This occurs because the first measure describes the amount of studies per connection, while the second shows the number of studies per edge. ‘Average degree’ and ‘average weighted degree’ are commonly used in social analyses to report positive and negative edges and its relationships [23, 25]. However, since NMA edges have no direction, we suggest the report of ‘median thickness’ (because it includes a dispersion measure), together with the report of the ‘percentage of strong edges’. These metrics seems more reasonable to calculate and interpret, and properly account for the weight of evidence in the network edges.

Besides the report of these parameters and metric of NMA geometry, the interpretation of the plots can also benefit from different design approaches. For instance, different colors for the edges to represent the level of confidence of comparisons between treatments (e.g. risk of bias) can be used. The amount of evidence can also be weighted in the nodes of the network-plots. Their sizes can proportionally represent the population sample included for each intervention [19]. However, this representation should be carefully evaluated since it can lead to inaccurate conclusions. The final size of a specific node should account for all of the samples of included studies on that specific intervention. There are several graphical tools available for drawing a network-plot and calculating geometric parameters and measures [52, 53]. Moreover, software such as R, STATA, or WinBUGS which are frequently used to perform the NMA statistics, can also be programmed to perform the diagrams and compute network metrics as well improve studies reporting [18, 27, 28, 54]. Additionally, authors’ of NMA should provide network graph for each outcome. The certainty of each treatment comparison should be estimated by using a standard approach like the GRADE (Grading of Recommendations Assessment, Development and Evaluation). To facilitate the visualization of the level of evidence (represented by the GRADE panels of outcome-graphs) or the risk of bias (Cochrane risk of bias assessment) different thickness or colors for individual edges should be used in NMA graphs [18, 31, 55, 56].

The main strength of our study was to suggest geometry metrics to standardize the report of NMA plot characteristics aiming at quantitative measure the NMA complexity, which may not be sufficiently evident just by the plot visual analysis. These metrics are simple and usable, both for technical and non-technical readers, and may guide further research on this topic. Our study also has some limitations. We included only NMAs of drug interventions and, although our results cannot not be immediately translated to other type of NMAs, there is nothing indicating differences among NMAs of different types of interventions. Further research on the relationships of network elements and other potential metrics of geometry should be explored. Bland-Altman limits of agreement are usually used to assess differences in normally distributed data; however, literature demonstrated that this test may be applied in non-normal data without a big impact [41, 42].

## Conclusions

Overall, seven simple geometry metrics were shown to be useful for describing NMA structure, contributing to data interpretation, and reproducibility. Guidelines and recommendations on the conduct and reporting of NMAs should strictly require the display of a network-plot and its complete description based on these metrics. Editors and peer-reviews should also ensure that reporting guidelines, including these items, are followed before publication.

## Supporting information

S1 File Complete search strategies. (DOCX)

S2 File Metrics to describe NMAs. (DOCX)

S3 File Bland-Altman plots and Lin’s concordance test. (DOCX)

S4 File PRISMA checklist. (DOC)

## References

[1] Kanters S, Ford N, Druyts E, Thorlund K, Mills EJ, Bansback N. Use of network meta-analysis in clinical guidelines. Bull World Health Organ. 2016;94(10):782–4. 10.2471/BLT.16.174326 27843171 PMC5043215

[2] Al Wattar BH, Zamora J, Khan KS. Informing treatment decisions through meta-analysis: to network or not? Evid Based Med. 2017;22(1):12–5. 10.1136/ebmed-2016-110599 27986815

[3] Bhatnagar N, Lakshmi PV, Jeyashree K. Multiple treatment and indirect treatment comparisons: An overview of network meta-analysis. Perspect Clin Res. 2014;5(4):154–8. 10.4103/2229-3485.140550 25276624 PMC4170532

[4] Tonin FS, Rotta I, Mendes AM, Pontarolo R. Network meta-analysis: a technique to gather evidence from direct and indirect comparisons. Pharm Pract (Granada). 2017;15(1):943 10.18549/PharmPract.2017.01.943 28503228 PMC5386629

[5] Dias S, Welton NJ, Caldwell DM, Ades AE. Checking consistency in mixed treatment comparison meta-analysis. Stat Med. 2010;29(7–8):932–44. Epub 2010/03/10. 10.1002/sim.3767 20213715

[6] Carroll K, Hemmings R. On the need for increased rigour and care in the conduct and interpretation of network meta-analyses in drug development. Pharm Stat. 2016;15(2):135–42. 10.1002/pst.1733 26732132

[7] Hutton B, Salanti G, Chaimani A, Caldwell DM, Schmid C, Thorlund K, et al The quality of reporting methods and results in network meta-analyses: an overview of reviews and suggestions for improvement. PLoS One. 2014;9(3):e92508 10.1371/journal.pone.0092508 24671099 PMC3966807

[8] Donegan S, Williamson P, D'Alessandro U, Tudur Smith C. Assessing key assumptions of network meta-analysis: a review of methods. Res Synth Methods. 2013;4(4):291–323. 10.1002/jrsm.1085 26053945

[9] Wang EK, Zou F. A new graph drawing scheme for social network. ScientificWorldJournal. 2014;2014:930314 10.1155/2014/930314 25157378 PMC4124209

[10] Dunn AG, Westbrook JI. Interpreting social network metrics in healthcare organisations: a review and guide to validating small networks. Soc Sci Med. 2011;72(7):1064–8. 10.1016/j.socscimed.2011.01.029 21371798

[11] Yousefi Nooraie R, Lohfeld L, Marin A, Hanneman R, Dobbins M. Informing the implementation of evidence-informed decision making interventions using a social network analysis perspective; a mixed-methods study. BMC Health Serv Res. 2017;17(1):122 10.1186/s12913-017-2067-9 28178958 PMC5299784

[12] Jia Y, Hoberock J, Garland M, Hart JC. On the visualization of social and other scale-free networks. IEEE Trans Vis Comput Graph. 2008;14(6):1285–92. 10.1109/TVCG.2008.151 18988975

[13] Gross JL, Yellen J, Zang P. Handbook of graph theory 2ed: Chapman & Hall/CRC; 2013.

[14] Scott J. Social Network Analysis. 3 ed London: SAGE; 2013.

[15] Rucker G. Network meta-analysis, electrical networks and graph theory. Res Synth Methods. 2012;3(4):312–24. 10.1002/jrsm.1058 26053424

[16] Hutton B, Salanti G, Caldwell DM, Chaimani A, Schmid CH, Cameron C, et al The PRISMA Extension Statement for Reporting of Systematic Reviews Incorporating Network Meta-analyses of Health Care Interventions: Checklist and Explanations. Ann Intern Med. 2015;162(11):777–84. 10.7326/M14-2385 26030634

[17] Cornell JE. The PRISMA extension for network meta-analysis: bringing clarity and guidance to the reporting of systematic reviews incorporating network meta-analyses. Ann Intern Med. 2015;162(11):797–8. 10.7326/M15-0930 26030637

[18] Chaimani A, Higgins JP, Mavridis D, Spyridonos P, Salanti G. Graphical tools for network meta-analysis in STATA. PLoS One. 2013;8(10):e76654 10.1371/journal.pone.0076654 24098547 PMC3789683

[19] Batson S, Score R, Sutton AJ. Three-dimensional evidence network plot system: covariate imbalances and effects in network meta-analysis explored using a new software tool. J Clin Epidemiol. 2017;86:182–95. 10.1016/j.jclinepi.2017.03.008 28344122

[20] Salanti G, Kavvoura FK, Ioannidis JP. Exploring the geometry of treatment networks. Ann Intern Med. 2008;148(7):544–53. 18378949 10.7326/0003-4819-148-7-200804010-00011

[21] Salanti G, Del Giovane C, Chaimani A, Caldwell DM, Higgins JP. Evaluating the quality of evidence from a network meta-analysis. PLoS One. 2014;9(7):e99682 10.1371/journal.pone.0099682 24992266 PMC4084629

[22] Anzures-Cabrera J, Higgins JP. Graphical displays for meta-analysis: An overview with suggestions for practice. Res Synth Methods. 2010;1(1):66–80. 10.1002/jrsm.6 26056093

[23] Arif T. The Mathematics of Social Network Analysis: Metrics for Academic Social Networks. International Journal of Computer Applications Technology and Research. 2015;4(12):889–93.

[24] Wasserman S, Faust K. Social Network Analysis: Methods and Applications: Cambridge: Cambridge University Press; 1994.

[25] Opsahl T, Agneessens F, Skvoretz J. Node centrality in weighted networks: Generalizing degree and shortest paths. Social Networks. 2010;32(245). 10.1016/j.socnet.2010.03.006

[26] Otte E, Rousseau R. Social network analysis: a powerful strategy, also for the information sciences. Journal of Information Science. 2002;28(6):441–53.

[27] Neupane B, Richer D, Bonner AJ, Kibret T, Beyene J. Network meta-analysis using R: a review of currently available automated packages. PLoS One. 2014;9(12):e115065 10.1371/journal.pone.0115065 25541687 PMC4277278

[28] Dias S, Sutton AJ, Ades AE, Welton NJ. Evidence synthesis for decision making 2: a generalized linear modeling framework for pairwise and network meta-analysis of randomized controlled trials. Med Decis Making. 2013;33(5):607–17. Epub 2012/10/30. 10.1177/0272989X12458724 23104435 PMC3704203

[29] Franchini AJ, Dias S, Ades AE, Jansen JP, Welton NJ. Accounting for correlation in network meta-analysis with multi-arm trials. Res Synth Methods. 2012;3(2):142–60. 10.1002/jrsm.1049 26062087

[30] Konig J, Krahn U, Binder H. Visualizing the flow of evidence in network meta-analysis and characterizing mixed treatment comparisons. Stat Med. 2013;32(30):5414–29. 10.1002/sim.6001 24123165

[31] Krahn U, Binder H, Konig J. A graphical tool for locating inconsistency in network meta-analyses. BMC Med Res Methodol. 2013;13:35 10.1186/1471-2288-13-35 23496991 PMC3644268

[32] Veroniki AA, Straus SE, Fyraridis A, Tricco AC. The rank-heat plot is a novel way to present the results from a network meta-analysis including multiple outcomes. J Clin Epidemiol. 2016;76:193–9. 10.1016/j.jclinepi.2016.02.016 26939929

[33] Mbuagbaw L, Rochwerg B, Jaeschke R, Heels-Andsell D, Alhazzani W, Thabane L, et al Approaches to interpreting and choosing the best treatments in network meta-analyses. Syst Rev. 2017;6(1):79 10.1186/s13643-017-0473-z 28403893 PMC5389085

[34] Sullivan SM, Coyle D, Wells G. What guidance are researchers given on how to present network meta-analyses to end-users such as policymakers and clinicians? A systematic review. PLoS One. 2014;9(12):e113277 10.1371/journal.pone.0113277 25517510 PMC4269433

[35] Lewis S, Clarke M. Forest plots: trying to see the wood and the trees. BMJ. 2001;322(7300):1479–80. 11408310 10.1136/bmj.322.7300.1479 PMC1120528

[36] Bax L, Ikeda N, Fukui N, Yaju Y, Tsuruta H, Moons KG. More than numbers: the power of graphs in meta-analysis. Am J Epidemiol. 2009;169(2):249–55. 10.1093/aje/kwn340 19064649

[37] Tan SH, Cooper NJ, Bujkiewicz S, Welton NJ, Caldwell DM, Sutton AJ. Novel presentational approaches were developed for reporting network meta-analysis. J Clin Epidemiol. 2014;67(6):672–80. 10.1016/j.jclinepi.2013.11.006 24560089

[38] Higgins JPT, Green S. Cochrane Handbook for Systematic Reviews of Interventions Version 5.1.0: Cochrane 2011.

[39] Moher D, Liberati A, Tetzlaff J, Altman DG. Preferred reporting items for systematic reviews and meta-analyses: the PRISMA statement. J Clin Epidemiol. 2009;62(10):1006–12. Epub 2009/07/28. 10.1016/j.jclinepi.2009.06.005 19631508

[40] Tonin FS, Borba HH, Leonart LP, Mendes AM, Steimbach LM, Pontarolo R, et al Methodological quality assessment of network meta-analysis of drug interventions: implications from a systematic review. Int J Epidemiol. 2018 10.1093/ije/dyy197 30212868

[41] Bland JM, Altman DG. Statistical methods for assessing agreement between two methods of clinical measurement. Lancet. 1986;1(8476):307–10. 2868172

[42] Giavarina D. Understanding Bland Altman analysis. Biochem Med (Zagreb). 2015;25(2):141–51. 10.11613/BM.2015.015 26110027 PMC4470095

[43] Lin LI. A concordance correlation coefficient to evaluate reproducibility. Biometrics. 1989;45(1):255–68. 2720055

[44] Senn S, Gavini F, Magrez D, Scheen A. Issues in performing a network meta-analysis. Stat Methods Med Res. 2013;22(2):169–89. 10.1177/0962280211432220 22218368

[45] Jansen JP, Naci H. Is network meta-analysis as valid as standard pairwise meta-analysis? It all depends on the distribution of effect modifiers. BMC Med. 2013;11:159 10.1186/1741-7015-11-159 23826681 PMC3707819

[46] Bafeta A, Trinquart L, Seror R, Ravaud P. Reporting of results from network meta-analyses: methodological systematic review. BMJ. 2014;348:g1741 10.1136/bmj.g1741 24618053 PMC3949412

[47] Zarin W, Veroniki AA, Nincic V, Vafaei A, Reynen E, Motiwala SS, et al Characteristics and knowledge synthesis approach for 456 network meta-analyses: a scoping review. BMC Med. 2017;15(1):3 10.1186/s12916-016-0764-6 28052774 PMC5215202

[48] Mavridis D, Giannatsi M, Cipriani A, Salanti G. A primer on network meta-analysis with emphasis on mental health. Evid Based Ment Health. 2015;18(2):40–6. 10.1136/eb-2015-102088 25908686 PMC11234956

[49] Cipriani A, Higgins JP, Geddes JR, Salanti G. Conceptual and technical challenges in network meta-analysis. Ann Intern Med. 2013;159(2):130–7. 10.7326/0003-4819-159-2-201307160-00008 23856683

[50] Cooper NJ, Peters J, Lai MC, Juni P, Wandel S, Palmer S, et al How valuable are multiple treatment comparison methods in evidence-based health-care evaluation? Value Health. 2011;14(2):371–80. 10.1016/j.jval.2010.09.001 21296599

[51] Sturtz S, Bender R. Unsolved issues of mixed treatment comparison meta-analysis: network size and inconsistency. Res Synth Methods. 2012;3(4):300–11. 10.1002/jrsm.1057 26053423

[52] Rucker G, Schwarzer G. Automated drawing of network plots in network meta-analysis. Res Synth Methods. 2016;7(1):94–107. 10.1002/jrsm.1143 26060934

[53] Chung H, Lumley T. Graphical exploration of network meta-analysis data: the use of multidimensional scaling. Clin Trials. 2008;5(4):301–7. 10.1177/1740774508093614 18697844

[54] Brown S, Hutton B, Clifford T, Coyle D, Grima D, Wells G, et al A Microsoft-Excel-based tool for running and critically appraising network meta-analyses—an overview and application of NetMetaXL. Syst Rev. 2014;3:110 10.1186/2046-4053-3-110 25267416 PMC4195340

[55] Brignardello-Petersen R, Murad MH, Walter SD, McLeod S, Carrasco-Labra A, Rochwerg B, et al GRADE approach to rate the certainty from a network meta-analysis: avoiding spurious judgments of imprecision in sparse networks. J Clin Epidemiol. 2019;105:60–7. 10.1016/j.jclinepi.2018.08.022 30253217

[56] Puhan MA, Schunemann HJ, Murad MH, Li T, Brignardello-Petersen R, Singh JA, et al A GRADE Working Group approach for rating the quality of treatment effect estimates from network meta-analysis. BMJ. 2014;349:g5630 10.1136/bmj.g5630 25252733

