# Supplementary material for: Description of network meta-analysis geometry: A metrics design study
Source: PLoS One. 2019 Feb 20;14(2):e0212650. doi: 10.1371/journal.pone.0212650 (PMC6382117; doi:10.1371/journal.pone.0212650)
Supplement: S1 File — (DOCX) [file pone.0212650.s001.docx]

**S1 File. Complete search strategy.**

| **PubMed** | "Bayesian meta-analysis" OR "network meta-analyses" OR "network meta-analysis" OR "Indirect meta-analyses" OR "Indirect meta-analysis" OR "Multiple treatment comparison" OR "Multiple treatment comparisons" OR "Multiple treatment meta-analysis" OR "Mixed treatment meta-analyses" OR "Mixed treatment meta-analysis" OR "Indirect treatment comparison" OR "Indirect treatment comparisons" (all fields) |
| --- | --- |
| **Scopus** | ( TITLE-ABS-KEY ( " Bayesian meta-analysis" OR "network meta-analyses" OR "network meta-analysis" OR "Indirect meta-analyses" OR "Indirect meta-analysis" OR "Multiple treatment comparison" OR "Multiple treatment comparisons" OR "Multiple treatment meta-analysis" OR "Mixed treatment meta-analyses" OR "Mixed treatment meta-analysis" OR "Indirect treatment comparison" OR "Indirect treatment comparisons")) |
