# Supplementary material for: Description of network meta-analysis geometry: A metrics design study
Source: PLoS One. 2019 Feb 20;14(2):e0212650. doi: 10.1371/journal.pone.0212650 (PMC6382117; doi:10.1371/journal.pone.0212650)
Supplement: S2 File — (DOCX) [file pone.0212650.s002.docx]

**S2 File. Metrics to describe NMAs.**

| **Metrics*** | **Formula** |
| --- | --- |
| **Graph** | $G_{NMA}=\left( N, E \right)$ |
| **Total degree** | $\sum\deg\left( N \right)=2\left\vert E \right\vert$ |
| **Average degree** | $D_{A}\left( G \right)=\frac{\sum deg(N)}{\left\vert N \right\vert}$  Also being expressed as:  $D_{A}\left( G \right)=\frac{2 \left\vert E \right\vert}{\left\vert N \right\vert}$ |
| **Average weighted degree** | ${WD}_{A}\left( G \right)= D_{A}\left( G \right)\times\frac{\left\vert Studies \right\vert}{\left\vert E \right\vert}==\frac{2 \left\vert E \right\vert}{\left\vert N \right\vert}\times\frac{\left\vert Studies \right\vert}{\left\vert E \right\vert}$  Which means:  ${WD}_{A}\left( G \right)=\frac{2\left\vert Studies \right\vert}{\left\vert N \right\vert}$ |
| **Density** | $D(G)=\frac{2\left\vert E \right\vert}{\left\vert N \right\vert(\left\vert N \right\vert-1)}$ |
| **Percentage common comparator**** | $CC(G)=\frac{N with>1 connection}{\left\vert N \right\vert}\times100$ |
| **Percentage strong edges**** | $E_{strong(G)}= \frac{E with>1 study}{\left\vert E \right\vert}\times100$ |
| **Mean thickness**** | $Mean thickness (G)=\frac{{WD}_{A}}{D_{A}}=\frac{\frac{2\left\vert Studies \right\vert}{\left\vert N \right\vert}}{\frac{2 \left\vert E \right\vert}{\left\vert N \right\vert}}$  Which means:  $Mean thickness \left( G \right)= \frac{Studies}{\left\vert E \right\vert}$ |
| **Median thickness**** | $Median of studies reported with IQR25 and IQR75$ |
| **Average path length** | $L(G)=\frac{1}{\left\vert N \right\vert(\left\vert N-1 \right\vert)}\times\sum_{i\neq j} d(N_{i},N_{j})$  ${d(N}_{i},N_{j}), where N_{i},N_{j}\in N denote the shortest distance between N_{i} and N_{j.}$ |
| N = number of nodes of a NMA;  E = number of edges of a NMA;  Studies = number of studies included in the NMA;  IQR: interquartile range  *All parameters and metrics were adapted from previous studies on social network analysis, graph theory (1-4). **Novel metrics especially created to support the report of NMAs geometry. | |

**References**

1. Opsahl T, Agneessens F, Skvoretz J**.** Node centrality in weighted networks: Generalizing degree and shortest paths. Social Networks. 2010;32(245).

2. Otte E, Rousseau R**.** Social network analysis: a powerful strategy, also for the information sciences. Journal of Information Science. 2002;28(6):441-53.

3. Wasserman S, Faust K**.** Social Network Analysis: Methods and Applications: Cambridge: Cambridge University Press; 1994.

4. Arif T**.** The Mathematics of Social Network Analysis: Metrics for Academic Social Networks. International Journal of Computer Applications Technology and Research. 2015;4(12):889-93.
