# Supplementary material for: Description of network meta-analysis geometry: A metrics design study
Source: PLoS One. 2019 Feb 20;14(2):e0212650. doi: 10.1371/journal.pone.0212650 (PMC6382117; doi:10.1371/journal.pone.0212650)
Supplement: S3 File — (DOCX) [file pone.0212650.s003.docx]

**S3 File. Bland-Altman plots and Lin’s concordance test.**

| **Metrics corresponding to ‘connections/node’ unit** | |
| --- | --- |
| **Average degree**  **Common comparators** | **Density**  **Common comparator** |
| 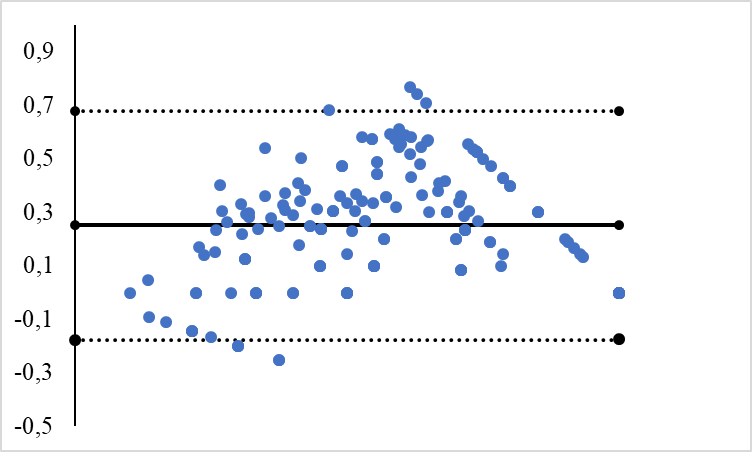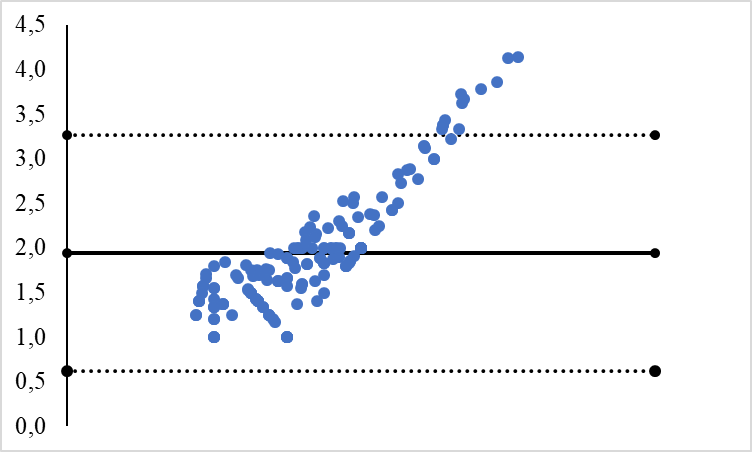 |  |
| Concordance coefficient ρc = 0.070  Lower one-sided 95% CL = 0.051  Lower two-sided 95% CL = 0.044  Upper one-sided 95% CL = 0.090  Upper two-sided 95% CL = 0.093 | Concordance coefficient ρc = 0.217  Lower one-sided 95% CL = 0.132  Lower two-sided 95% CL = 0.116  Upper one-sided 95% CL = 0.298  Upper two-sided 95% CL = 0.313 |

**S3 File. Bland-Altman plots and Lin’s concordance test.**

| **Metrics corresponding to ‘studies/edge’ unit** | |
| --- | --- |
| **Average weighted degree**  **Mean thickness** | **Average weighted degree**  **Median thickness** |
| 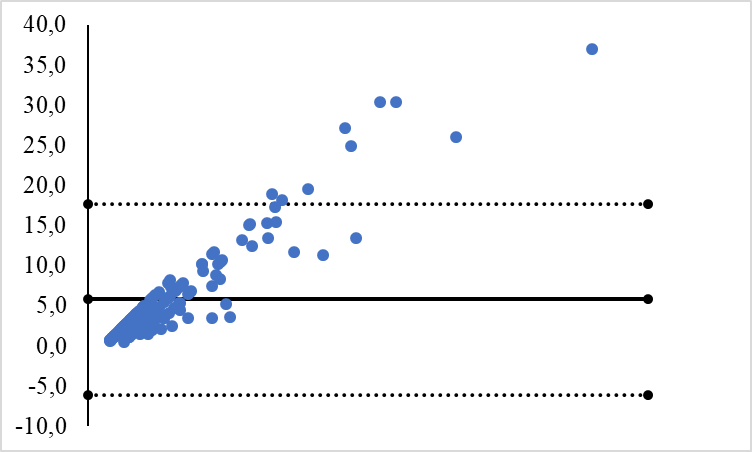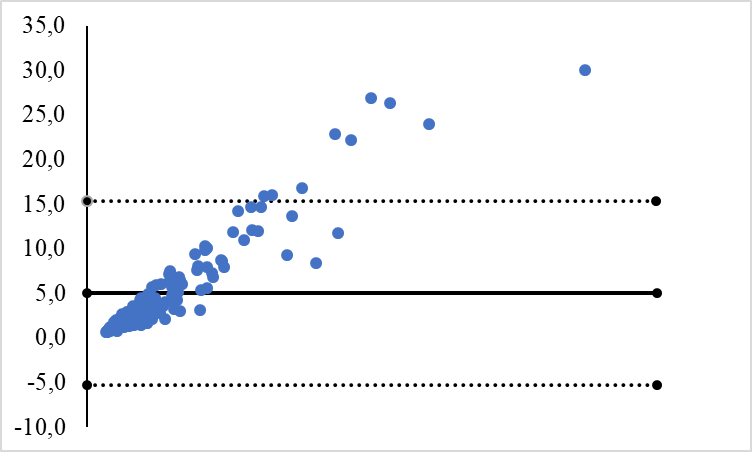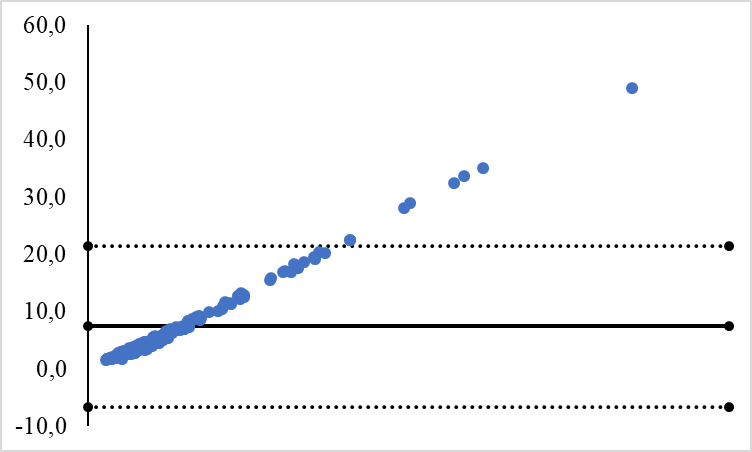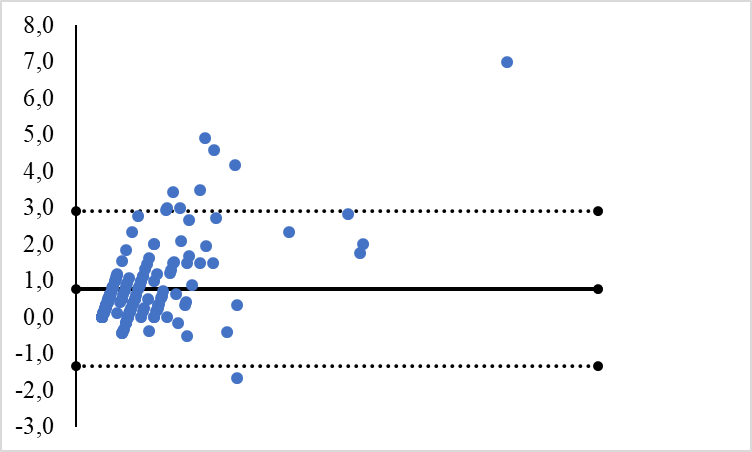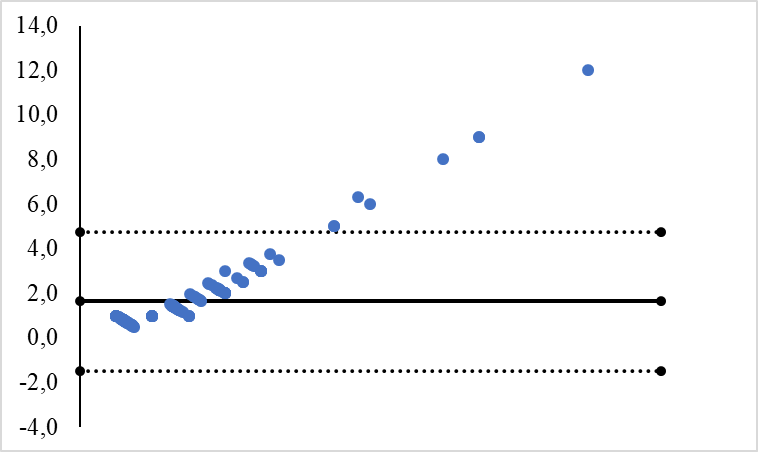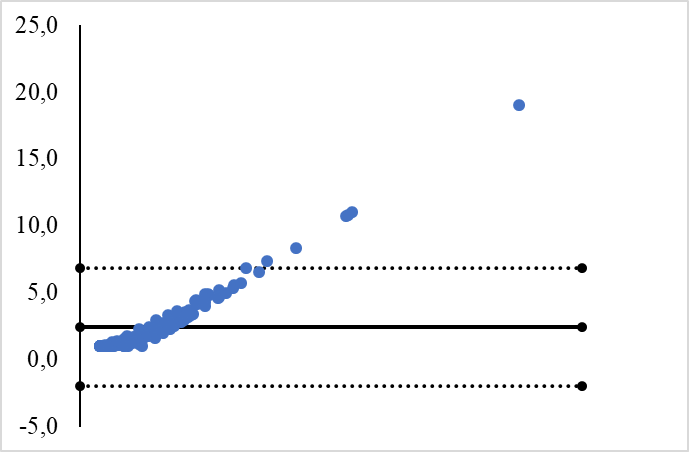 |  |
| Concordance coefficient ρc = 0.367  Lower one-sided 95% CL = 0.323  Lower two-sided 95% CL = 0.315  Upper one-sided 95% CL = 0.410  Upper two-sided 95% CL = 0.418 | Concordance coefficient ρc = 0.220  Lower one-sided 95% CL = 0.181  Lower two-sided 95% CL = 0.174  Upper one-sided 95% CL = 0.252  Upper two-sided 95% CL = 0.259 |
| **Average weighted degree**  **Strong edges** | **Mean thickness**  **Median thickness** |
|  |  |
| Concordance coefficient ρc = 0.031  Lower one-sided 95% CL = 0.022  Lower two-sided 95% CL = 0.020  Upper one-sided 95% CL = 0.040  Upper two-sided 95% CL = 0.042 | Concordance coefficient ρc = 0.820  Lower one-sided 95% CL = 0.780  Lower two-sided 95% CL = 0.772  Upper one-sided 95% CL = 0.850  Upper two-sided 95% CL = 0.855 |
| **Strong edges**  **Mean thickness** | **Strong edges**  **Median thickness** |
|  |  |
| Concordance coefficient ρc = 0.103  Lower one-sided 95% CL = 0.080  Lower two-sided 95% CL = 0.071  Upper one-sided 95% CL = 0.129  Upper two-sided 95% CL = 0.134 | Concordance coefficient ρc = 0.170  Lower one-sided 95% CL = 0.128  Lower two-sided 95% CL = 0.121  Upper one-sided 95% CL = 0.203  Upper two-sided 95% CL = 0.209 |
